# Feasibility and Acceptability of a Physical Activity Tracker and Text Messages to Promote Physical Activity During Chemotherapy for Colorectal Cancer: Pilot Randomized Controlled Trial (Smart Pace II)

**DOI:** 10.2196/31576

**Published:** 2022-01-11

**Authors:** Erin L Van Blarigan, Anand Dhruva, Chloe E Atreya, Stacey A Kenfield, June M Chan, Alexandra Milloy, Iris Kim, Paige Steiding, Angela Laffan, Li Zhang, Sorbarikor Piawah, Yoshimi Fukuoka, Christine Miaskowski, Frederick M Hecht, Mi-Ok Kim, Alan P Venook, Katherine Van Loon

**Affiliations:** 1 Department of Epidemiology and Biostatistics University of California, San Francisco San Francisco, CA United States; 2 Department of Urology University of California, San Francisco San Francisco, CA United States; 3 Osher Center for Integrative Medicine University of California, San Francisco San Francisco, CA United States; 4 Department of Medicine University of California, San Francisco San Francisco, CA United States; 5 Helen Diller Family Comprehensive Cancer Center University of California, San Francisco San Francisco, CA United States; 6 University of California, Berkeley Berkeley, CA United States; 7 School of Nursing University of California, San Francisco San Francisco, CA United States

**Keywords:** exercise, treatment, colon cancer, rectal cancer, digital health, wearables, SMS

## Abstract

**Background:**

We conducted a pilot 2-arm randomized controlled trial to assess the feasibility of a digital health intervention to increase moderate-to-vigorous physical activity in patients with colorectal cancer (CRC) during chemotherapy.

**Objective:**

This study aimed to determine whether a digital health physical activity intervention is feasible and acceptable during chemotherapy for CRC.

**Methods:**

Potentially eligible patients with CRC expected to receive at least 12 weeks of chemotherapy were identified in person at the University of California, San Francisco, and on the web through advertising. Eligible patients were randomized 1:1 to a 12-week intervention (Fitbit Flex, automated SMS text messages) versus usual care. At 0 and 12 weeks, patients wore an Actigraph GT3X+ accelerometer for 7 days and completed surveys, body size measurements, and an optional 6-minute walk test. Participants could not be masked to their intervention arm, but people assessing the body size and 6-minute walk test outcomes were masked. The primary outcomes were adherence (eg, Fitbit wear and text response rate) and self-assessed acceptability of the intervention. The intervention would be considered feasible if we observed at least 80% complete follow-up and 70% adherence and satisfaction, a priori.

**Results:**

From 2018 to 2020, we screened 240 patients; 53.3% (128/240) of patients were ineligible and 26.7% (64/240) declined to participate. A total of 44 patients (44/240, 18%) were randomized to the intervention (n=22) or control (n=22) groups. Of these, 57% (25/44) were women; 68% (30/44) identified as White and 25% (11/44) identified as Asian American or Pacific Islander; and 77% (34/44) had a 4-year college degree. The median age at enrollment was 54 years (IQR 45-62 years). Follow-up at 12 weeks was 91% (40/44) complete. In the intervention arm, patients wore Fitbit devices on a median of 67 out of 84 (80%) study days and responded to a median of 17 out of 27 (63%) questions sent via SMS text message. Among 19 out of 22 (86%) intervention patients who completed the feedback survey, 89% (17/19) were satisfied with the Fitbit device; 63% (12/19) were satisfied with the SMS text messages; 68% (13/19) said the SMS text messages motivated them to exercise; 74% (14/19) said the frequency of SMS text messages (1-3 days) was ideal; and 79% (15/19) said that receiving SMS text messages in the morning and evening was ideal.

**Conclusions:**

This pilot study demonstrated that many people receiving chemotherapy for CRC are interested in participating in digital health physical activity interventions. Fitbit adherence was high; however, participants indicated a desire for more tailored SMS text message content. Studies with more socioeconomically diverse patients with CRC are required.

**Trial Registration:**

ClinicalTrials.gov NCT03524716; https://clinicaltrials.gov/ct2/show/NCT03524716

## Introduction

### Background

Colorectal cancer (CRC) is the fourth most diagnosed cancer and the second leading cause of cancer-related deaths in the United States [1]. Prospective studies suggest that physical activity after CRC diagnosis is associated with longer survival, including in patients with advanced or metastatic disease [2-5]. Moreover, patients with nonmetastatic CRC who engage in less physical activity after diagnosis have a 32% increased risk of CRC-specific mortality compared with patients who maintain their prediagnosis levels of activity [6]. Given that physical activity tends to decline during treatment [7], interventions that help patients with CRC to maintain their physical activity levels during treatment may be important adjuncts to standard oncological therapies.

Several interventions are being evaluated for their impact on physical activity in patients with CRC [8]. The Colon Health and Life-Long Exercise Change (CHALLENGE) and Focus on Reducing Dose-limiting Toxicities in Colon Cancer with Resistance Exercise (FORCE) trials are 2 examples of such interventions [8,9]. CHALLENGE is an active randomized controlled trial examining the effects of a structured exercise program on disease-free survival among patients with high-risk stage 2 or 3 colon cancer who have completed adjuvant chemotherapy [8]. FORCE is an open randomized controlled trial examining the effects of resistance training on relative dose intensity and chemotoxicities in patients with nonmetastatic colon cancer receiving adjuvant chemotherapy [9]. Notably, CHALLENGE was designed as a supervised program, and FORCE focused on resistance training; both studies enrolled only patients with nonmetastatic colon cancer. Indeed, most studies to date have focused on people with nonmetastatic disease and those who have already completed treatment. There remains a need to determine the feasibility of physical activity interventions for patients with CRC during active treatment. Moreover, participation in supervised exercise intervention programs for patients with cancer may be limited by time, expense, and access to treatment centers offering exercise services. Thus, remotely delivered interventions may increase the accessibility of exercise interventions for patients with CRC.

### Previous Work

Digital health tools, such as physical activity trackers, SMS text messaging, and apps, offer low-cost approaches to increase physical activity [10]. One study evaluated adherence to wearing a Fitbit in patients with early breast cancer on chemotherapy and concluded that additional intervention components, such as phone calls, SMS text messages, or other reminders, are needed to maintain adherence to wearing the Fitbit [11]. Few studies have evaluated similar intervention components in patients with CRC, especially those undergoing chemotherapy. A review of consumer wearable health intervention studies with survivors of breast cancer, prostate cancer, and CRC identified 8 randomized controlled trials conducted among people with these cancers; only one of these trials (Smart Pace I), conducted by our team, focused exclusively on survivors of CRC [12]. In that study, we reported that digital health tools, including a Fitbit Flex and SMS text messages, were feasible, were acceptable, and may increase physical activity among survivors of CRC after completion of chemotherapy [13].

### Objectives

In this study (Smart Pace II), we aim to determine whether a digital health physical activity intervention is feasible and acceptable during chemotherapy, with the goal to prevent the decline in physical activity that often occurs during treatment for CRC. We conducted a 12-week pilot 2-arm randomized controlled trial with patients with CRC receiving chemotherapy. Our primary objective is to evaluate the feasibility and acceptability of a digital physical activity intervention in this patient population. In addition, we sought to estimate the effect of the intervention on physical activity, cardiorespiratory fitness estimated through the 6-minute walk test distance, body weight, and blood pressure from enrollment to 12 weeks.

## Methods

Smart Pace II was a 2-arm (1:1) pilot randomized controlled trial. The study was approved by the institutional review board of the University of California, San Francisco (UCSF).

### Study Population and Recruitment

#### Overview

We recruited individuals with colon or rectal cancer who were recommended to receive at least 12 weeks of chemotherapy. Potentially eligible participants were identified through the Gastrointestinal Oncology Clinic at UCSF as well as through public advertising on the web, at community events, and in local oncology clinics. Potential participants at the UCSF were approached in person and by email. The intervention was administered remotely, and recruitment was not restricted to individuals receiving chemotherapy at the UCSF. Eligibility criteria included the expectation of receiving at least 12 weeks of chemotherapy, the ability to speak and read English, access to a mobile phone with email and SMS text messaging capabilities, ≥4 weeks since the last major surgery, and provider endorsement of patient safety to participate in unsupervised moderate physical activity. Patients were excluded if they self-reported ≥150 minutes per week of moderate-to-vigorous physical activity (MVPA) on the modified Godin Leisure Time Exercise Questionnaire or had contraindications to exercise at the time of enrollment [14]. We initially excluded participants who owned a physical activity tracker designed to be worn all day (not just during exercise sessions), such as a Fitbit. In June 2019, we refined this criterion to exclude people who owned physical activity trackers and had worn them in the past month; people who owned trackers but were not wearing them would still be eligible. The eligibility criterion that excluded people who owned and wore a physical activity tracker was completely removed in August 2019.

**Figure 1:**
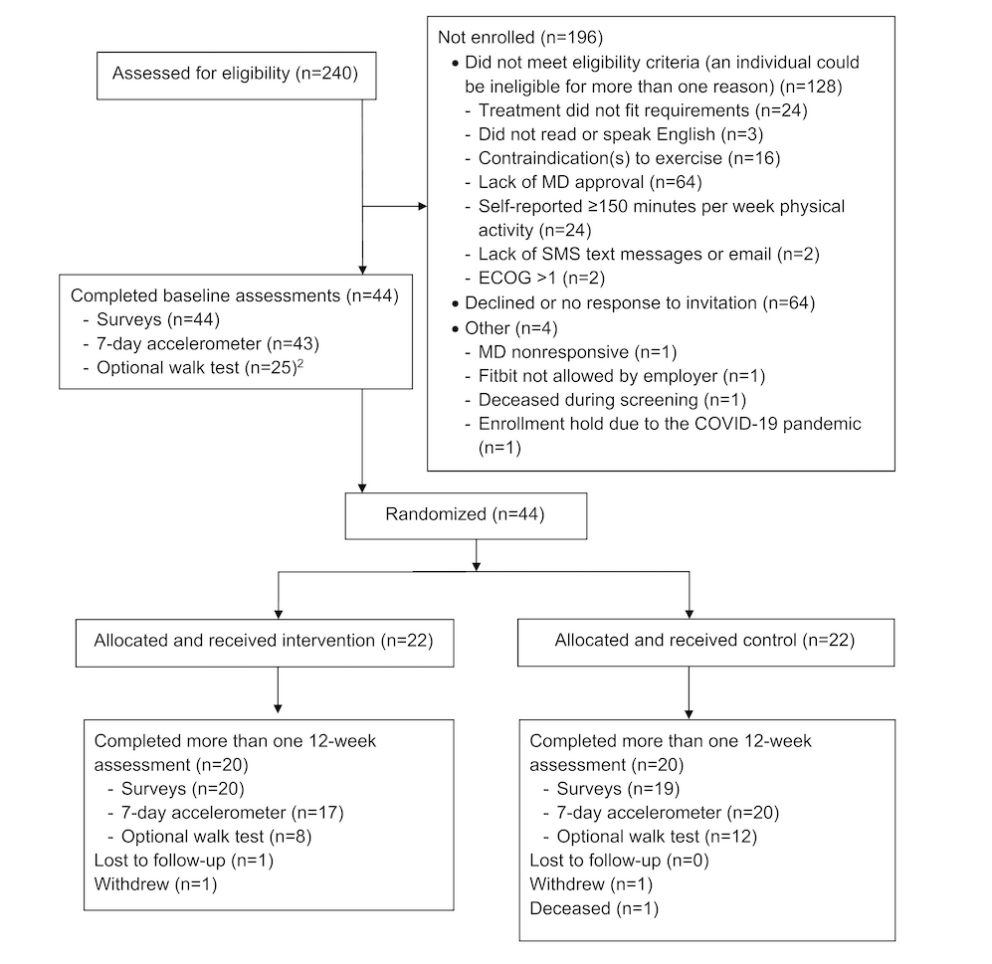
CONSORT (Consolidated Standards of Reporting Trials) flow diagram for the Smart Pace II study, a randomized controlled pilot study evaluating a 12-week physical activity intervention for people receiving chemotherapy for colon or rectal cancer. Stay Home Public Orders were enacted on March 17, 2020, in San Francisco, California, and all elective medical visits were cancelled, including two baseline and five 12-week 6-minute walk tests. ECOG: Eastern Cooperative Oncology Group; MD: medical doctor.

Between March 1, 2018, and March 17, 2020, a total of 240 patients were assessed for eligibility (Figure 1). Of these 240 people, 26.7% (64/240) declined to participate. Interested patients were asked to complete a web-based screening survey using Research Electronic Data Capture (REDCap) [15]. We then contacted the treating provider for each potential participant to confirm clinical eligibility and endorsement of the patient’s safety to engage in unsupervised moderate physical activity. One provider did not respond, so we were unable to ascertain eligibility for one potential participant. Following these screening procedures, 53.8% (128/240) of the patients were deemed ineligible. The main reasons were lack of provider approval (64/128, 50%), a treatment plan that did not match the eligibility requirements (24/128, 18.8%), medical contraindications to exercise (16/128, 12.5%), or self-reported exercise of ≥150 minutes per week of MVPA (24/128, 18.8%). One patient passed away during the screening period and one patient was not allowed to wear the Fitbit at work. Thus, after recruitment and screening, 18.8% (45/240) of screened patients were considered eligible for participation.

#### Consent and Randomization

Once participants were confirmed as eligible, informed consent was obtained either in person or electronically using DocuSign. Between March 15, 2018, and March 20, 2020, a total of 44 participants were randomized 1:1 to intervention or control, using a computer-generated randomization scheme created by a blinded study statistician (LZ). The 45th interested and eligible participant was not randomized owing to an enrollment hold as a result of the COVID-19 pandemic. The randomization scheme was uploaded to REDCap, and the study research coordinator used REDCap to determine a given participant’s assigned intervention arm. Relevant study materials were then distributed to the participants in person or by mail by the study research coordinator.

### Interventions

#### Intervention Arm

Participants in the intervention arm received a printed booklet about physical activity after cancer, daily fully automated SMS text messages (see Multimedia Appendix 1 for sample SMS text messages), a Fitbit Flex 2 Fitness Wristband (hereafter referred to as the Fitbit), and a list of home-based exercise apps and videos. The intervention was intended to be stand-alone with no human involvement. Participants received written instructions on how to set up the Fitbit and were asked to wear their Fitbit on their wrist every day during the 12-week study period; they were allowed to keep the Fitbit after the study. To receive the SMS text messages automatically during the study, participants’ phone numbers were registered by a research coordinator on a custom-built Drupal website that interacted with Twilio to facilitate sending and receiving SMS text messages. Participants were encouraged to work up to the United States Physical Activity Guidelines of 150 minutes per week of MVPA through the SMS text messages [16]. A total of 21 SMS text messages specifically promoted aerobic exercise, 10 specifically mentioned resistance exercise, and 2 SMS text messages specifically encouraged flexibility exercise. Notably, 4 SMS text messages asked participants, “Good Morning! How is your energy level today? Text back ‘H’ if you feel great, ‘M’ if you feel ok, and ‘L’ if you feel very tired.” Tailored feedback for the day’s activity was sent based on the participants’ responses. For example, if the participant replied “L,” they received the following message: “(1/2) You are going through a lot. Sometimes light exercise can help you feel better. (2/2) Walking or yoga are good options—try to do just 10 minutes today at an easy and comfortable pace and see if that helps!” A total of 6 SMS text messages prompted the participants to wear and synchronize their Fitbit devices. Owing to the nature of the intervention, the participants were not blinded to their assigned intervention arm.

#### Control Arm

Participants in the control arm received a printed booklet about physical activity for cancer survivors after randomization and were given a Fitbit after completion of the 12-week follow-up assessments to compensate for study participation.

### Study Measures

#### Feasibility

We assessed the feasibility of the intervention by calculating the median number of days that intervention participants wore the Fitbit; the median number of SMS text messages that asked for a reply that intervention participants responded to; and the proportion of the study participants who completed at least one 12-week follow-up survey, overall and by arm. We counted the Fitbit as worn on a given day if >1500 steps were recorded [17]. SMS text message adherence was calculated as the mean proportion of texts that requested a reply to which each intervention participant responded. We stated that we would consider the intervention to be feasible if we achieved at least 70% adherence on average (Fitbit worn at least 59 days out of the 84 study days; 19 or more text messages responded to out of 27 that asked for a reply) and if 80% of participants completed at least one 12-week follow-up survey, a priori.

#### Acceptability

The acceptability of the intervention was evaluated by an investigator-created questionnaire administered at 12 weeks on the web using REDCap [15]. Intervention participants were asked to what degree they agreed with statements regarding the intervention components (eg, SMS text messages and Fitbit). Responses were coded on a 5-point Likert scale (eg, 1=strongly agree, 2=agree, 3=undecided, 4=disagree, and 5=strongly disagree). The questionnaire also included 2 open-ended questions for other feedback on the SMS text messages and Fitbit devices.

#### Physical Activity

Participants’ physical activity was assessed as a secondary outcome. Activity was measured using ActiGraph GTX3+ accelerometers (ActiGraph LLC) worn on the wrist for 7 consecutive days at enrollment and 12 weeks [18]. Data were recorded and analyzed in 5-second epochs. A minimum of 3 days with a valid wear time of at least 10 hours was required for inclusion in the analysis [19,20]. To determine valid hours, nonwear time was identified using the Troiano 2007 algorithm in the ActiLife software (version 6.13.4).

After the study was completed, we used the Freedson Adult 1998 cutoff points to identify the average minutes per day of sedentary (0-100 counts per minute), light (101-1952 counts per minute), moderate (1953-5724 counts per minute), hard (5725-9498 counts per minute), and very hard (9499-16,000 counts per minute) physical activity [21]. We also estimated minutes per week spent in at least 10-minute bouts of MVPA. To do so, we divided the total time in Freedson Adult 1998 bouts calculated by the ActiLife software by the number of calendar days with valid wear time and multiplied by 7. These calculations were performed after the study was completed, so participants and researchers were blinded to the baseline accelerometer-assessed physical activity minutes per week values at the time of randomization.

#### 6-Minute Walk Test, Body Weight, and Blood Pressure

At enrollment and 12 weeks, participants who were able to come to the UCSF were given the option to complete a 6-minute walk test, a submaximal test correlated with peak VO_2_ and widely used to detect changes in exercise tolerance in adults [22]. If the test was performed on the same day as the scheduled treatment, the 6-minute walk test was performed before the administration of chemotherapy. Data on participants’ body weight and blood pressure were abstracted from participants’ medical records (patients from UCSF) or obtained from participants’ providers (patients not from UCSF) at baseline and 12 weeks.

#### Adverse Events

A survey was created by the investigator team to collect self-reported adverse events during the intervention period. Participants completed a brief *health check-in* on the web at 0, 4, 8, and 12 weeks using REDCap surveys delivered via email. The survey queried recent chemotherapy treatments, current body weight, medication use, hospitalizations, and whether the patient had experienced any of the following conditions in the past 4 weeks: low back pain, knee pain, shoulder pain, arthritis, chest pain, shortness of breath, fatigue, leg cramping, muscle pain, and dizziness or vertigo. If participants reported any of these conditions, they were asked to report the onset and duration of symptoms, whether any activities made it better or worse, and if they took any medication for the condition.

#### Sample Size

Our target sample size of 48 participants was based on the number of participants in previous pilot studies [13]. This number was sufficient to answer our primary objective of feasibility, quantified using Fitbit adherence (number of days that the participants wore the device) and text message response (number of replies to SMS text messages that asked for a reply). We stopped the trial in March 2020, after 44 participants were randomized, owing to the COVID-19 pandemic.

### Statistical Analysis

Descriptive statistics, including counts, percentages, means, SDs, medians, and ranges were used to describe participant characteristics and reports of adverse events. All statistical analyses were conducted using R [23].

We conducted 1-sample *Z* tests to determine whether the observed adherence was significantly less than the a priori cutoff of 70%. We also used 1-sample *Z* tests to determine whether the proportion of the study participants (overall and by group) that completed a 12-week follow-up survey was significantly less than a prior cutoff of 80% or more*.* Fisher exact test was used to compare attrition between the 2 arms. We reported the participants’ responses to the feedback questionnaire using descriptive statistics.

The secondary effects of the intervention from baseline to 12 weeks within and between the intervention and control arms were estimated using weighted *t* tests for physical activity measures and Mann–Whitney tests for body weight, blood pressure, and the 6-minute walk test.

## Results

We randomized 44 participants with CRC to the intervention (n=22) or control (n=22) arms (Figure 1) between March 2018 and March 2020. The assigned intervention was administered to all 44 participants. Follow-up at 12 weeks was 91% (20/22) complete in both arms. In the intervention arm, one participant withdrew, reporting that the study was incompatible with the chemotherapy schedule and citing the inconvenience of charging and syncing the Fitbit. One intervention arm patient was lost to follow-up for unknown reasons. In the control arm, 1 participant died during the intervention phase because of cancer progression, and 1 participant withdrew after transferring care to another treatment facility.

### Study Population Characteristics

The characteristics of the intervention and control arms are listed in Table 1. Most participants (29/44, 66%) were enrolled at the start of their first line of chemotherapy, and 6 were receiving their third or more line of chemotherapy (6/44, 14%). The individuals enrolled with an initial diagnosis of stage 1 or 2 disease were receiving neoadjuvant chemotherapy (1 person), adjuvant chemotherapy to reduce the risk of recurrence (3 people), or chemotherapy for recurrent disease (1 person). The intervention and control groups had a similar median age at enrollment and similar gender and cancer site, stage, and treatment distributions. However, by chance owing to the small sample size, a higher proportion of the control group were patients from UCSF who identified as Asian American or Pacific Islander; the median BMI of this group was also lower than that of the intervention group.

**Table 1:** Demographic characteristics and clinical factors of participants with colorectal cancer undergoing chemotherapy in a 2-arm pilot randomized controlled trial of a 12-week digital physical activity intervention (N=44).

Characteristics		Total	Intervention	Control
Participants, n (%)		44 (100)	22 (50)	22 (50)
Patients from UCSF^a^, n (%)		37 (84)	15 (34)	22 (50)
Age at enrollment (years), median (IQR)		54 (45-62)	53 (41-59)	53 (47-67)
Females, n (%)		25 (57)	14 (32)	11 (25)
BMI (kg/m^2^), median (IQR)		25.7 (21.5-28.7)	27.5 (22.7-30.5)	24.0 (20.8-26.9)
**Highest level of education, n (%)**				
	2-year college or less	10 (23)	6 (14)	4 (9)
	4-year college	16 (36)	9 (20)	7 (16)
	Graduate or professional degree	18 (41)	7 (16)	11 (25)
**Self-identified race or origin^b^, n (%)**				
	Asian American or Pacific Islander	11 (25)	3 (7)	8 (18)
	White	30 (68)	18 (41)	12 (27)
	Other or unknown	4 (9)	2 (5)	2 (5)
**Primary cancer site, n (%)**				
	Colon	28 (64)	12 (27)	16 (36)
	Rectum	16 (36)	10 (23)	6 (14)
Months since diagnosis, median (IQR)		4 (2-19)	4 (2-6)	4 (2-8)
**Stage at diagnosis, n (%)**				
	1-2	5 (11)	3 (7)	2 (5)
	3	22 (50)	10 (23)	12 (27)
	4	17 (39)	9 (21)	8 (18)
**Treatments received for colon or rectal cancer at the time of enrollment (all that apply), n (%)**				
	Surgery	28 (64)	14 (32)	14 (32)
	Radiation	5 (11)	3 (7)	2 (5)
	Systemic chemotherapy	44 (100)	22 (50)	22 (50)
	Other	1 (2)	0	1 (2)
**Ostomy status at enrollment, n (%)**				
	No ostomy	32 (73)	16 (36)	16 (36)
	Permanent ostomy	6 (14)	4 (9)	2 (5)
	Previously reversed ostomy	2 (5)	1 (2)	1 (2)
	Ostomy awaiting reversal	4 (9)	1 (2)	3 (7)
**Current line of chemotherapy, n (%)**				
	1	29 (66)	13 (30)	16 (36)
	2	9 (20)	4 (9)	5 (11)
	≥3	6 (14)	5 (11)	1 (2)
**Disease status at enrollment, n (%)**				
	No evidence of disease	5 (11)	1 (2)	4 (9)
	Stable disease	18 (41)	10 (23)	8 (18)
	Progressive disease	21 (48)	11 (25)	10 (23)
**Smoking status, n (%)**				
	Never	30 (68)	13 (30)	17 (39)
	Former	14 (32)	9 (20)	5 (11)
Comorbidities^b^, median (IQR)		1 (0-2)	1 (1-1)	1 (1-1)
**Comorbid conditions^b^, n (%)**				
	High blood pressure	13 (30)	5 (11)	8 (18)
	Elevated cholesterol	13 (30)	6 (14)	7 (16)
	Cancer (not including CRC^c^)	7 (16)	5 (11)	2 (5)
	Arthritis	5 (11)	3 (7)	2 (5)
	Diabetes mellitus	4 (9)	1 (2)	3 (7)
	Venous thromboembolism	4 (9)	2 (5)	2 (5)
	Chronic kidney disease	3 (7)	1 (2)	2 (5)
	Asthma	2 (5)	1 (2)	1 (2)
	Other comorbid conditions^d^	6 (14)	2 (5)	4 (9)

^a^UCSF: University of California, San Francisco.^b^Comorbid conditions were ascertained using self-report.

^c^CRC: colorectal cancer.

^d^Other comorbidities reported by 1 person each included transient ischemic attack, stroke, osteoporosis, history of hip fracture, multiple sclerosis, emphysema, or chronic bronchitis.

### Adherence and Attrition

**Figure 2:**
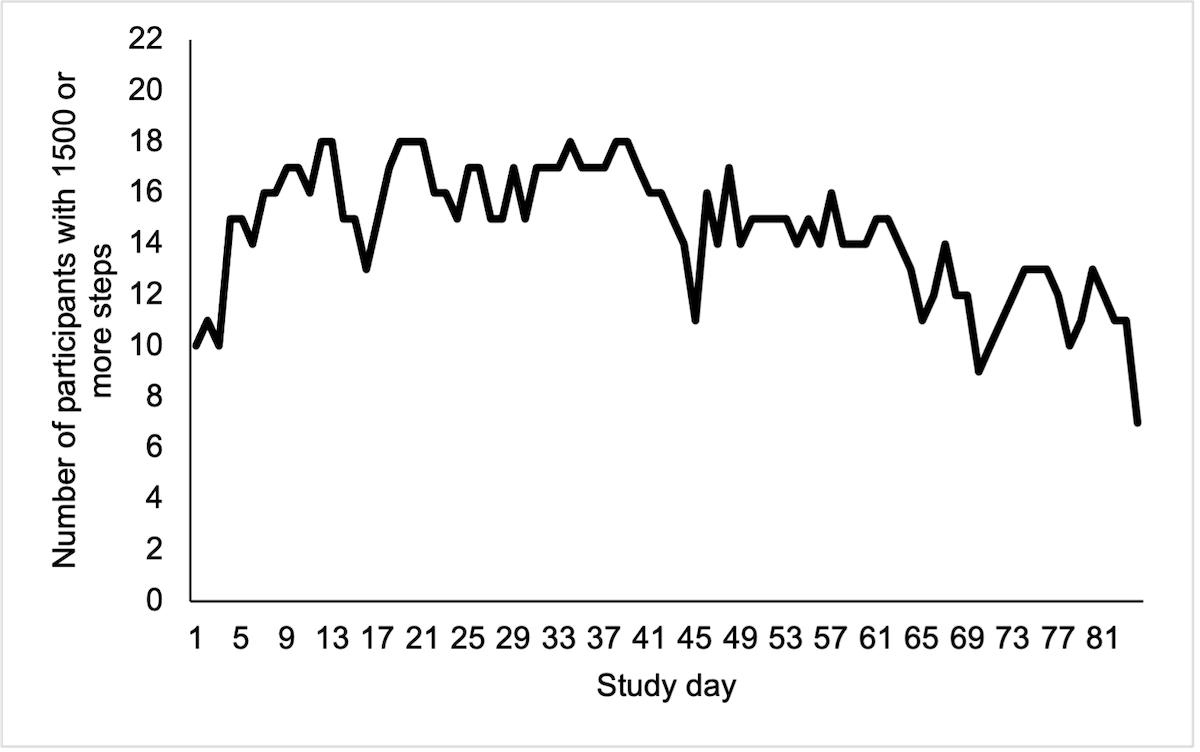
Number of participants in the intervention arm of the Smart Pace II pilot study who recorded at least 1500 steps per day on the Fitbit, by study day (n=22).

Participants randomized to the intervention arm wore their Fitbits for a median of 67 out of 84 study days (IQR 53-80 days). A total of 2 participants never wore the Fitbit, and 2 participants had <10 days of wear time. A total of 6 participants had >80 days of wear time. Fitbit use trended down slightly over time (Figure 2). There was no correlation between age and gender of the participants and wear time. Participants with stage 4 cancer had a median Fitbit wear time of 56 days (IQR 47-76 days) compared with a median of 77 days among participants with stage 1 to 3 disease (IQR 56-82).

**Figure 3:**
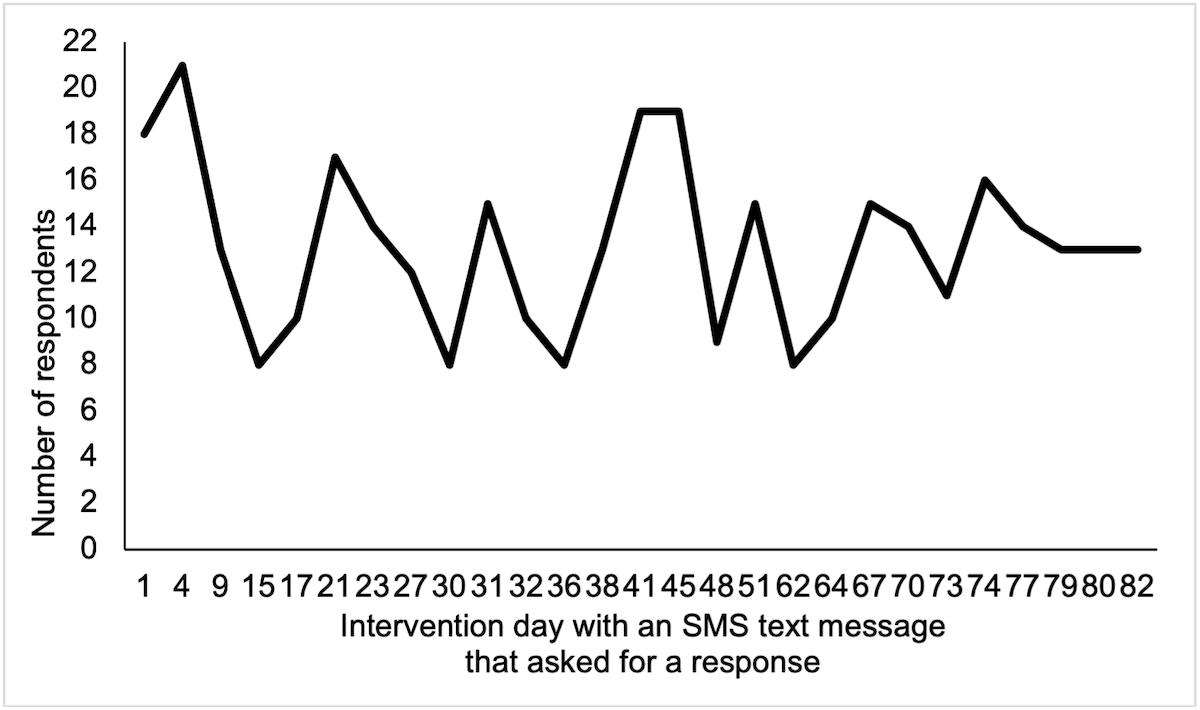
Number of participants in the intervention arm who responded to the SMS text messages that asked for a reply in the Smart Pace II pilot study (n=22).

Overall, participants in the intervention arm responded to a median of 17 out of 27 SMS text messages that asked for a reply (63%; IQR 12-23; range 1-26). SMS text message response rates fluctuated over time (Figure 3). SMS text messages sent on days 15, 36, and 62, which queried whether participants had achieved the goals they were asked to set at the beginning of the study on day 8, were among the messages with the lowest response rates. No patterns were observed regarding the content of SMS text messages that received the highest response rates. SMS text message response rates did not vary by age, gender, or cancer stage.

### Acceptability

Most participants reported that the intervention was acceptable (Table 2 and Table 3). Out of the 22 participants in the intervention arm, 19 (86%) completed the feedback questionnaire. Among the respondents, 63% (12/19) reported satisfaction with the SMS text messages overall and 89% (17/19) reported satisfaction with the Fitbit and an expectation that they would continue to wear the Fitbit after the study ended.

**Table 2:** Overall satisfaction with 12 weeks of SMS text messages and a Fitbit Flex 2 among individuals receiving chemotherapy for colorectal cancer (n=22).

Responses	Very satisfied	Satisfied	Neutral	Dissatisfied	Very dissatisfied	Missing
Overall satisfaction with text messages, n (%)	4 (18)	8 (36)	6 (27)	1 (5)	0	3 (14)
Overall satisfaction with Fitbit, n (%)	7 (32)	10 (46)	1 (5)	1 (5)	0	3 (14)

When asked about specific features (Table 3), 68% (13/19) agreed that the SMS text messages motivated them to exercise and that the content was interesting; 74% (14/19) said that the frequency of the messages was ideal (1 every 1-3 days), and 79% (15/19) said that the timing of the messages was ideal (morning and evening). The most frequent recommendation for improvement was to improve the personalization of messages (Multimedia Appendix 2). Regarding the Fitbit, 2 participants said that they did not like using a wearable device or did not feel the need to track their activities daily. Additional feedback from participants included difficulty adhering to the intervention because of treatment-related fatigue and restrictions imposed during the COVID-19 pandemic.

**Table 3:** Responses to the feedback survey regarding acceptability of 12 weeks of SMS text messages and a Fitbit Flex 2 among individuals receiving chemotherapy for colorectal cancer (n=22 participants).

Responses	Strongly agree	Agree	Undecided	Disagree	Strongly disagree	Missing
Text messages motivated me to exercise, n (%)	3 (14)	10 (46)	1 (5)	4 (18)	1 (5)	3 (14)
Content of text messages was interesting, n (%)	3 (14)	10 (46)	3 (14)	1 (5)	2 (9)	3 (14)
Frequency of text messages was ideal, n (%)	6 (27)	8 (36)	2 (9)	1 (5)	2 (9)	3 (14)
Timing of text messages was ideal, n (%)	3 (14)	12 (55)	2 (9)	1 (5)	1 (5)	3 (14)
Fitbit motivated me to exercise, n (%)	7 (32)	9 (41)	1 (5)	2 (9)	0 (0)	3 (14)

### Estimated Changes in Physical Activity

Physical activity levels measured by the accelerometer at enrollment and 12 weeks for participants in the intervention and control groups are shown in Multimedia Appendix 3. No patient recorded any vigorous physical activity at any time point. Overall, on average, the participants accumulated 110 minutes per week (SD 103 minutes per week) of moderate-intensity activity in bouts of 10 minutes or longer at enrollment. By chance, the intervention arm recorded more time in moderate activity bouts compared with controls at enrollment (mean 141.5, SD 115.5 minutes per week and mean 80.7, SD 83.5 minutes per week, respectively).

When examining changes from 0 to 12 weeks, both the intervention and control groups decreased their physical activity on average over the 12-week study period. The intervention arm had a mean reduction in moderate activity accumulated in 10-minute bouts of 21.3 minutes per week (SD 144.8 minutes per week); the control arm had a mean reduction in moderate activity accumulated in 10-minute bouts of 16.3 minutes per week (SD 121.2 minutes per week). There was no difference in the change in moderate activity accumulated in bouts of 10 minutes when comparing the 2 groups (mean difference 0.2, SD 6.2 minutes per week). When examining individual changes, 47% (8/17) of participants in the intervention arm and 35% (7/20) in the control arm with data at both time points increased bouts of moderate activity from enrollment to 12 weeks by at least 1 minute. Notably, when examining total activity throughout the day (not specifically ≥10-minute bouts), participants in the intervention arm reduced moderate activity, light activity, and steps more than the control arm. Finally, the total time moving at a moderate intensity was high in both the intervention and control arms and the change in activity between time points was highly variable with wide SDs.

### Estimated Changes in 6-Minute Walk Test, Body Weight, and Blood Pressure

The 6-minute walk test, body weight, and blood pressure at enrollment and 12 weeks for participants in the intervention and control groups are shown in Multimedia Appendix 3. Participants in both arms increased their 6-minute walk test distance by an average of 37 meters (SD 39 meters) in the intervention group and 46 meters (SD 59 meters) in the control group. For body weight, the intervention group had a mean change of −0.8 pounds (SD 5.7 pounds), whereas the control group had a mean change of 0.1 pounds (SD 9.5 pounds). When examining individual changes, we observed that 65% (11/17) participants in the intervention arm with data available at both time points lost weight from enrollment to 12 weeks, whereas 42% (8/19) participants in the control arm lost weight from 0 to 12 weeks. There were no significant changes in blood pressure within or between the 2 groups. The average difference in systolic blood pressure from 0 to 12 weeks for the intervention group was 6.4 mm Hg (SD 12.2 mm Hg); and the control group had a mean change of −0.4 mm Hg (SD 10.6 mm Hg). The average difference in diastolic blood pressure for the intervention group was −0.5 mm Hg (SD 9.6 mm Hg); the control group had a mean change of −5.8 mm Hg (SD 9.0 mmm Hg). When examining individual changes, 53% (9/17) participants in the intervention arm and 67% (12/18) participants in the control arm decreased their systolic and diastolic blood pressure from 0 to 12 weeks. As with the physical activity data, there was considerable variability in responses between participants.

### Adverse Events

The number of reported adverse events is presented in Table 4*.* There were no serious adverse events related to the intervention, and the intervention did not appear to increase reports of nonserious adverse events compared with baseline. A total of 4 participants in the control group reported hospitalizations during the study, and 1 participant in the control group passed away during the study because of cancer progression. There were no hospitalizations or deaths in the intervention group.

For nonserious adverse events, fatigue was the most reported adverse event during the study, but the number of times fatigue was reported was highest at enrollment and it did not increase during the intervention period. In addition, as described above, 4 of the SMS text messages in the intervention arm asked participants to rate how they felt (days 4, 31, 45, and 67). On day 4, 32% (7/22) of the participants in the intervention arm responded saying they were very tired, 27% (4/15) said they were very tired on day 31, 11% (2/19) said they were very tired on day 45, and 27% (4/15) said they were very tired on day 67.

**Table 4:** Adverse events reported among participants receiving chemotherapy and participating in a 12-week digital physical activity intervention^a^ (N=44).

Adverse events	Intervention, n (%)					Control, n (%)			
	Before enrollment (n=22)	0-4 weeks (n=17)	5-8 weeks (n=20)	9-12 weeks (n=19)	Before enrollment (n=22)		0-4 weeks (n=17)	5-8 weeks (n=15)	9-12 weeks (n=19)
Total adverse events	51	40	48	38	38		31	20	22
Low back pain	7 (14)	4 (10)	5 (10)	6 (16)	4 (11)		1 (3)	1 (5)	2 (9)
Knee pain	2 (4)	2 (5)	2 (4)	1 (3)	1 (3)		0 (0)	0 (0)	1 (5)
Shoulder pain	3 (6)	2 (5)	5 (10)	2 (5)	4 (11)		1 (3)	0 (0)	0 (0)
Inflammation of the joints	2 (4)	2 (5)	2 (4)	0 (0)	1 (3)		2 (6)	1 (5)	1 (5)
Chest pain	0 (0)	1 (3)	2 (4)	1 (3)	1 (3)		0 (0)	0 (0)	0 (0)
Shortness of breath	5 (10)	3 (8)	4 (8)	5 (13)	2 (5)		0 (0)	0 (0)	1 (5)
Fatigue	18 (35)	13 (33)	16 (33)	14 (37)	12 (32)		13 (42)	11 (55)	12 (55)
Leg cramping	4 (8)	3 (8)	2 (4)	1 (3)	2 (5)		4 (13)	1 (5)	1 (5)
Muscle pain	4 (8)	3 (8)	4 (8)	2 (5)	2 (5)		3 (10)	1 (5)	0 (0)
Dizziness or vertigo	2 (4)	1 (3)	3 (6)	2 (5)	2 (5)		2 (6)	1 (5)	1 (5)
Other orthopedic limitation	1 (2)	2 (5)	0 (0)	0 (0)	1 (3)		2 (6)	2 (10)	1 (5)
Doctor’s visit, excluding standard cancer follow-up	3 (6)	4 (10)	3 (6)	4 (11)	3 (8)		2 (6)	2 (10)	1 (5)
Hospitalization^b^	0 (0)	0 (0)	0 (0)	0 (0)	3 (8)		1 (3)	0 (0)	0 (0)
Death^c^	N/A^d^	0 (0)	0 (0)	0 (0)	N/A		0 (0)	0 (0)	1 (5)

^a^Participants were asked at the time of enrollment to report if they had experienced any adverse events in the past month. The survey was repeated at 4, 8, and 12 weeks.

^b^Reasons for hospitalization in the month before enrollment included anemia, infection, and fever after receipt of chemotherapy, and at 0-4 weeks, stomach perforation.

^c^One participant in the control arm expired while enrolled in the study because of cancer progression.

^d^N/A: not applicable.

## Discussion

### Principal Findings

Overall, we observed that a remotely delivered physical activity intervention that included a wristband for self-monitoring physical activity and SMS text messages during chemotherapy for CRC was feasible and acceptable. Although this study was not powered to detect changes in physical activity, our pilot data show a nonstatistically significant decrease in moderate activity accumulated in bouts of at least 10 minutes in both arms (16-21 minutes per week).

### Comparison With Previous Work

Notably, the findings from this study with participants who were actively receiving chemotherapy differed from our previous study in people who had previously completed treatment for CRC. In our previous study (Smart Pace I), we observed an average increase in physical activity in participants in the intervention arm [13]. The main difference in our SMS text message content for this study (Smart Pace II) was the addition of questions about how participants felt and tailored activity advice in response. This modification was based on our expectation that participants would feel fatigued during chemotherapy and need support or motivation to promote activity. Messaging to *take it easy* or *build up slowly* sent to a group of people who were active at baseline and felt tired on treatment may have unintentionally contributed to why the intervention arm decreased activity levels slightly more than the control arm, which did not receive SMS text messaging. Further research is required to evaluate whether such messages would have the intended beneficial effect in a sedentary population (encouraging those who feel tired to do a light activity vs nothing). In addition, delivering more nuanced messages that encourage active people to stay active during treatment even when they are tired, without pushing them too far, is a challenge for automated intervention approaches such as SMS text messaging.

Few other studies have conducted remote physical activity interventions in patients with CRC or survivors. Kim et al [24] reported that a home-based exercise intervention with weekly supervised components (counseling or training sessions) significantly increased self-reported moderate physical activity from 97 minutes per week at enrollment to 325 minutes per week at 12 weeks, with no change observed among the controls. These data are consistent with previous findings from our team in a study of men with prostate cancer. In the Community of Wellness study, we observed a modest change in self-reported physical activity but only among men who reported <90 minutes per week of activity at baseline and in the group that received one coaching call with an exercise trainer [25]. It is possible that these previous studies reported greater changes in activity compared with this pilot study because they used self-report rather than objective measures. Nonetheless, some degree of coaching or more personalized contact may be needed to help people with cancer assess their current level of activity and identify what changes are needed to meet the physical activity guidelines and optimize their cancer outcomes.

### Limitations

The baseline physical activity level measured at enrollment was high in both arms and particularly high in the intervention arm. This occurred despite the exclusion of prospective participants who self-reported ≥150 minutes per week of MVPA. However, physical activity measured using the accelerometers indicated that self-reported MVPA may have underestimated actual MVPA. It is also possible that participants engaged in higher than usual levels of activity when wearing the devices. Interestingly, when we analyzed moderate activity accumulated in bouts of ≥10 minutes, the participants’ activity levels were similar to self-report. Although logistically difficult, future studies should consider using accelerometer data to determine eligibility or set a lower cutoff point for self-report to ensure they enroll an inactive study population who may most benefit from the intervention.

In addition, our sample included highly educated participants and low enrollment of Black or Latinx CRC survivors, which may limit the generalizability of our findings. Given the high CRC incidence and mortality among Black people and rising rates of young-onset CRC in some Latinx populations, research is critically needed in these patient groups [26,27]. Self-identified race or ethnicity was not assessed in our study until after participants provided consent. Although Hispanic or Latinx patients comprise 17% of patients with CRC at our institution, it is possible many may have been excluded owing to the requirement for English proficiency. We encourage future studies to support translations into multiple languages and to track the race or ethnicity of all screened participants to identify and address potential barriers to enrollment and ensure future studies enroll representative patient populations.

Finally, the COVID-19 pandemic began while the last 7 participants were active in the study. Several of our SMS text messages provided tips for participants to find social support and exercise with others, which were perceived by participants as irrelevant or incompatible with social distancing guidelines imposed during the pandemic. Out of these 7 participants, 6 (85%) participants had paired accelerometer data available. Out of these 6 participants, 5 (83%) decreased their time spent in bouts of moderate activity at 12 weeks compared with enrollment; 1 participant increased their time spent in moderate activity bouts. Although the numbers are small, it is possible that the pandemic led to a slightly greater decrease in planned moderate activity from enrollment to 12 weeks in our study, on average, than would have been observed in a study conducted before the COVID-19 pandemic. Decreases in physical activity, on average, have also been reported among noncancer study populations during the pandemic [28].

### Future Work

Although the intervention was determined to be feasible and acceptable, there are aspects that could be improved in future studies. The main takeaway based on participant feedback was that the intervention, specifically the SMS text messages, needed to be more personalized. For example, several participants suggested that SMS text messages could be tailored to the data collected from the Fitbit. Future studies may be strengthened by having real-time access to activity data to determine appropriate messaging. Machine learning approaches, such as reinforcement learning, could also provide a platform to improve the tailoring of SMS text messages [29]. In addition, 4 participants in the intervention arm did not wear the Fitbit. Although the number is small, it is worth considering offering other mechanisms for self-monitoring physical activity, such as paper diaries, in future studies. Finally, studies are needed to determine the feasibility and acceptability of digital health physical activity interventions in individuals with lower levels of education, individuals with low English proficiency, and individuals who identify with minority racial or ethnic groups. Adaptation of digital health interventions and messaging into other languages and with attention to cultural contexts will be critical to improving access for a more diverse population.

### Conclusions

Overall, this pilot study demonstrated that patients with CRC were interested in a remotely delivered, automated digital health physical activity intervention during chemotherapy. However, more tailored support is needed to further enhance participant satisfaction and possibly improve physical activity behavior.

## Appendix

Multimedia Appendix 1 Sample text messages from the Smart Pace II study.

Multimedia Appendix 2 Participant feedback on the intervention components collected at 12 weeks using an open text write-in field in the participant feedback survey.

Multimedia Appendix 3 Mean physical activity, 6-minute walk test, body weight, and blood pressure at baseline and 12-week among participants in a 12-week pilot randomized controlled trial of a Fitbit Flex 2 and daily text messages.

Multimedia Appendix 4 CONSORT-eHEALTH checklist (V 1.6.1).

## Abbreviations

CHALLENGE: The Colon Health and Life-Long Exercise Change
CRC: colorectal cancer
FORCE: Focus on Reducing Dose-limiting Toxicities in Colon Cancer with Resistance Exercise
MVPA: moderate-to-vigorous physical activity
REDCap: Research Electronic Data Capture
UCSF: University of California, San Francisco
