# Supplementary material for: Feasibility and Acceptability of a Physical Activity Tracker and Text Messages to Promote Physical Activity During Chemotherapy for Colorectal Cancer: Pilot Randomized Controlled Trial (Smart Pace II)
Source: JMIR Cancer. 2022 Jan 11;8(1):e31576. doi: 10.2196/31576 (PMC8790683; doi:10.2196/31576)
Supplement: Multimedia Appendix 1 [file cancer_v8i1e31576_app1.docx]

| Multimedia Appendix 1. First two weeks of text messages sent to participants in the intervention arm of the Smart Pace II randomized controlled trial. |
| --- |
| Day 1 |
| "(1/4) Welcome to the Smart Pace II Study, brought to you by the UCSF Gastrointestinal  Oncology Program and the Osher Center for Integrative Medicine."  "(2/4) Each day, you'll receive a text message. We may ask for a short reply. Text STOP at any time if you no longer want to receive our messages."  "(3/4) If you have any questions, call us at: 415-514-6314 or email us at: smartpacestudy@ucsf.edu"  "(4/4) If you feel ill or get injured, call your doctor or 911. Please text back 'Y' to confirm that you received this text." |
| Day 2 |
| "Wear your Fitbit & sync it with your computer or phone to keep track of your activity. Double tap the display to see your progress toward your daily goal." |
| Day 3 |
| "(1/2) The American Cancer Society recommends 150 minutes per week of physical activity. Exercise lasting 10 or more minutes counts toward your weekly total!"  "(2/2) Be active throughout your day by taking the stairs, parking farther away from your destination,  or doing squats while watching TV.” |
| Day 4 |
| "Good morning! How is your energy level today? Text back 'H' if you feel great, 'M' if you feel ok, and 'L' if you feel very tired." |
| Day 5 |
| "(1/2) 150 minutes of exercise per week may sound like a lot, but you don't have to do it all at once."  "(2/2) Spread out your exercise during the week. You can break it into small chunks of just 10-15 minutes at a time." |
| Day 6 |
| "Take a moment to think about reasons why you have not been physically active in the past. Then write down ideas for how to get past what's stopping you." |
| Day 7 |
| "If you have not been active for a while, start at a comfortable level and add time and intensity  gradually. Exercising with a friend may help you get started." |
| Day 8 |
| "(1/2) Take a moment to write down exercise goals for next week, next month, and three months from now. Keep these handy so you can refer to them often."  "(2/2) It might seem tedious in the moment, but writing down your goals is very helpful for meeting your goals in the long run." |
| Day 9 |
| "How about a walk after lunch or dinner today? If you can do this, text back 'Y'. If not, text back 'N'." |
| Day 10 |
| "Do workouts that are enjoyable to you. Note how it feels to do them and how good it feels afterward - doing things we truly enjoy is very motivating." |
| Day 11 |
| "Wear your Fitbit every day to capture all the activities that you do! Keeping track of your physical activity will help you achieve your exercise goals." |
| Day 12 |
| "Short on time? Start with 10 minutes at a time, a couple of days a week. Walk during a break. Dance in your living room. It all adds up." |
| Day 13 |
| "Looking for different ways to be active? Try hiking, gardening, golf (walking between holes), bicycling, swimming. Choose something you love, and do it often!" |
| Day 14 |
| "(1/2) Don't let others stand in YOUR way of pursuing good health.",  "(2/2) Tell your family and friends that you are serious about exercise. Maybe you will motivate them to exercise too!" |
