# Supplementary material for: Feasibility and Acceptability of a Physical Activity Tracker and Text Messages to Promote Physical Activity During Chemotherapy for Colorectal Cancer: Pilot Randomized Controlled Trial (Smart Pace II)
Source: JMIR Cancer. 2022 Jan 11;8(1):e31576. doi: 10.2196/31576 (PMC8790683; doi:10.2196/31576)
Supplement: Multimedia Appendix 2 [file cancer_v8i1e31576_app2.docx]

| Multimedia Appendix 2. Participant feedback from a 12-week digital physical activity intervention for colorectal cancer patients on chemotherapy. |
| --- |
| *Text messages* |
| “It was very motivational and kept me in track.” |
| "I thought messages that were specific to cancer patients were most useful and relevant. Especially ones that referenced survival benefits of exercise." |
| "They seemed very generic instead of being more focused to the individual receiving the text message. It might be more helpful to have text messages come through that correspond to result that populate via Fitbit or that are more catered to the individual. Some of the texts coming though also seemed outdated and a little depressive when mentioning how ‘physical activity may lower risk of death by 30% in people with current stage III colon cancer (2013)...’ etc." |
| "Well, that is good news. So aim for 150+ minutes per week. Maybe I can ask [my doctor] to explain more what the numbers mean. More exercise gives you a better chance of living longer. I get that. But it's 28% of what? That's an answer that's tough to get. The more interesting question for me is: If one's longevity is estimated at 65% chance of living 3 years, then walking for 150 minutes per week gives you a 28% chance of living another 3 months? Is that right. Or does the 65% chance get reduced by 28% so I have a 47% of living 3 years. But I definitely get the message. I only mention this to you because as researchers you should know what your patients think about when they receive messages like this. It does motivate you to exercise more. That's good. And living longer is good. But it also reminds patients that their lives will be ending decade(s) earlier than intended. Not so good.” (Patient’s response to the Day 16 text message: *Among >4500 colorectal cancer patients, walking approx. 150 min/wk after diagnosis was associated with a 28% lower risk of death (Schmid Ann Oncol 2014).*) |
| "I felt like the texts received were too frequent for their relevancy. If the texts were more catered to the individual, then they would be more effective. Maybe having it geared toward individual Fitbit statistics to inquire about their daily steps, etc." |
| "I prefer weekly messages myself as it allows me to plan for the week and visualize how exercise fits into my schedule." |
| *Fitbit* |
| "The Fitbit maintains consistency of keeping conscious of your activity level and therefore present with your movements (or lack thereof), which in turns helps keep your exercise and activity level top of mind." |
| "The idea of using a wearable to keep track of my exercises didn't appeal to me." |
| "I don't feel the need to track daily activity. I naturally stay active when I'm not sick (it’s cold season & my resistance is unusually low due to infusions). Many people like to track their activity and check their Fitbit/tracking device often." |
| "Being able to track my steps motivated me to make sure I got a minimum. However, there were some days I was too exhausted. The longer I do chemotherapy, the more tired and longer the side effects last. It also helped me try to get longer hours sleeping. I did notice the Fitbit tracked ‘steps’ that were really from rocking in a rocking chair. I didn't sit in one and rock after noticing it." |
| *Barriers* |
| "I had a lot of personal changes come up during the program that I think I could've followed it better otherwise." |
| "I was too tired to take advantage of all the exercise apps. It needed to be simpler." |
| "I felt some of the messages didn't take into consideration that some people are still recovering from abdominal surgery and can't do some of those things suggested due to not wanting to develop a hernia. Also, due to the shelter in place, there were more restrictions about getting out to do some activities." |
| "During pandemic...some advice was irrelevant e.g., going to gym, exercising with friends... and my fatigue was so bad I could not consider the suggestion ...feeling too bad" |
