# Supplementary material for: Feasibility and Acceptability of a Physical Activity Tracker and Text Messages to Promote Physical Activity During Chemotherapy for Colorectal Cancer: Pilot Randomized Controlled Trial (Smart Pace II)
Source: JMIR Cancer. 2022 Jan 11;8(1):e31576. doi: 10.2196/31576 (PMC8790683; doi:10.2196/31576)
Supplement: Multimedia Appendix 3 [file cancer_v8i1e31576_app3.docx]

| **Multimedia Appendix 3**. Mean physical activity, 6-minute walk test, body weight, and blood pressure at baseline and 12-week among participants in a 12-week pilot randomized controlled trial of a Fitbit Flex 2 and daily text messages. | | | | | | | |
| --- | --- | --- | --- | --- | --- | --- | --- |
|  | **Intervention** | | | **Control** | | |  |
|  | **Baseline^1^** | **12 Weeks** | **Mean change ±SD** | **Baseline** | **12 Weeks** | **Mean change ±SD** | **Mean difference in change between groups ±SD^2^** |
| **Physical Activity^3^** | | | | | | | |
| No. of participants | 21 | 18 | 17 | 22 | 20 | 20 | 17 Intervention  20 Control |
| Time in bouts of moderate activity, minutes per week^4^ | 141.5 ± 115.5 | 172.6 ± 131.6 | -21.3 ± 144.8 | 80.7 ± 83.5 | 70.2 ± 87.9 | -16.3 ± 121.2 | 0.2 ± 6.2 |
| Vigorous, min/d | 0 ± 0 | 0 ± 0 | 0 ± 0 | 0 ± 0 | 0 ± 0 | 0 ± 0 | 0 ± 0 |
| Moderate, min/d | 152.3 ± 40.0 | 146.1 ± 51.4 | -17.4 ± 52.8 | 121.0 ± 42.0 | 116.9 ± 54.2 | -8.3 ± 55.6 | -6.9 ± 17.3 |
| Light, min/d | 229.2 ± 51.1 | 203.5 ± 53.6 | -33.8 ± 56.8 | 202.6 ± 47.2 | 194.2 ± 66.0 | -12.4 ± 56 | -20.2 ± 17.7 |
| Steps, min/d | 9376 ± 1971 | 8736 ± 3115 | -1139 ± 3058 | 7472 ± 2402 | 7268 ± 3091 | -431 ± 2993 | -641 ± 956 |
| **6-minute walk test** | | | | | | | |
| No. of participants | 11 | 8 | 8 | 14 | 12 | 10 | 8 Intervention  10 Control |
| Distance, m | 462.5 ± 86.8 | 517.1 ± 48.4 | 37.1 ± 38.9 | 453.3 ± 106 | 499.1 ± 74.5 | 45.8 ± 58.8 | -8.7± 70.5 |
| **Body weight** | | | | | | | |
| No. of participants | 21 | 17 | 17 | 22 | 19 | 19 | 17 Intervention  19 Control |
| Weight, lb | 183.0 ±60.5 | 183.5 ±56.8 | -0.8 ±5.7 | 157.3 ±53.5 | 153.3 ±45.0 | 0.1 ±9.5 | -0.9 ±11.1 |
| **Blood pressure** | | | | | | | |
| No. of participants | 21 | 17 | 17 | 22 | 18 | 18 | 17 Intervention  18 Control |
| Systolic (mmHg) | 121.0 ±13.3 | 128.7 ±13.4 | 6.4 ±12.2 | 124.8 ±16.6 | 121.6 ±15.2 | -0.4 ±10.6 | 6.8 ±16.2 |
| Diastolic (mmHg) | 73.4 ±10.8 | 73.7 ±11.5 | -0.5 ±9.6 | 76.5 ±9.9 | 70.9 ±7.2 | -5.8 ±9.0 | 5.4 ±13.2 |

Abbreviations: SD, standard deviation; min, minute; d, day.

^1^ One participant’s accelerometer at baseline was lost in the mail.

^2^ Comparing change in intervention group minus change in control group.

^3^ Physical activity was measured using a wrist-worn Actigraph GT3X+ accelerometer (5-second epochs).

^4^ A bout of moderate activity was defined lasting 10 minutes or more, estimated using the Freedson 1998 bouts in the Actilife software.
